# Respiratory Infection- and Asthma-prone, Low Vaccine Responder Children Demonstrate Distinct Mononuclear Cell DNA Methylation Pathways

**DOI:** 10.21203/rs.3.rs-4160354/v1

**Published:** 2024-04-03

**Authors:** David Martino, Nikki Schultz, Ravinder Kaur, Simon D. Haren, Nina Kresoje, Annmarie Hoch, Joann Diray-Arce, Jessica Lasky Su, Ofer Levy, Michael Pichichero

**Affiliations:** University of Western Australia; University of Western Australia; Rochester General Hospital; Boston Children’s Hospital; University of Western Australia; Boston Children’s Hospital; Boston Children’s Hospital; Harvard Medical School; Boston Children’s Hospital; Rochester General Hospital

## Abstract

**Background:**

Infants with frequent viral and bacterial respiratory infections exhibit compromised immunity to routine immunisations. They are also more likely to develop chronic respiratory diseases in later childhood. This study investigated the feasibility of epigenetic profiling to reveal endotype-specific molecular pathways with potential for early identification and immuno-modulation. Peripharal immune cells from respiratory infection allergy/asthma prone (IAP) infants were retrospectively selected for genome-wide DNA methylation and single nucleotide polymorphism analysis. The IAP infants were enriched for the low vaccine responsiveness (LVR) phenotype (Fishers Exact p-value = 0.01).

**Results:**

An endotype signature of 813 differentially methylated regions (DMRs) comprising 238 lead CpG associations (FDR < 0.05) emerged, implicating pathways related to asthma, mucin production, antigen presentation and inflammasome activation. Allelic variation explained only a minor portion of this signature. Stimulation of mononuclear cells with monophosphoryl lipid A (MPLA), a TLR agonist, partially reversing this signature at a subset of CpGs, suggesting the potential for epigenetic remodelling.

**Conclusions:**

This proof-of-concept study establishes a foundation for precision endotyping of IAP children and highlights the potential for immune modulation strategies using adjuvants for furture investigation.

## Background

Respiratory infections in early life are among the leading causes of childhood morbidity and mortality and the most common cause of respiratory admissions to hospitals (1). While vaccines can help prevent some respiratory diseases, some children exhibit a phenotype of low responsiveness to their routine schedule of vaccines (2), and are vulnerable to upper respiratory tract infections, particularly acute otitis media (3, 4). A 10-year study of acute otitis media (AOM) children conducted in Rochester, New York, revealed that children prone to AOM, termed otitis prone (OP), exhibit sub-protective IgG antibody levels to most of their scheduled paediatric vaccine antigens (5). Clinically, they also experience substantially higher rates of subsequent allergies and asthma (6). Otitis prone children are more susceptible to respiratory infections due to associated nasopharyngeal (NP) and systemic immune deficits (7, 8). Specifically, a reduced capacity to respond to infections is associated with reduced activation of Toll-like receptor (TLR) signalling pathways and cytokine production (8), leading to attenuated stimulation of CD4^+^ T cell responses, specifically Th17 responses to extracellular pathogens (9). This disparity in immune development in children who suffer from high rates of otitis-media, allergy and asthma constitutes a unique endotype of ‘respiratory infection, allergy/asthma-prone children’ (IAP) that merits focused investigation for the development of precision interventions (2, 9–11).

Endotyping approaches to classify diseases based on molecular and biological pathways, rather than clinical symptoms, have the potential to lead to new options for early preventative treatments (12, 13). In this study we undertook a precision endotyping approach focused on DNA methylation biomarkers (CpG methylation) which are regulatory base modifications to DNA that influence cellular immune responses across the life course (14). Such modifications vary according to host genotypic variation and environmental influences, and therefore contain information about both genome and environment (15). Their potential utility for endotyping is supported by studies that demonstrate respiratory infections modify host CpG methylation in both nasal tissue and blood leukocytes (16, 17).

Post-infectious epigenetic modifications to leukocyte DNA can be retained at immune-response genes, a concept known as ‘epigenetic scars’, and these modifications can induce cellular non-responsiveness (‘tolerance’), increasing host susceptibility to severe infections and sepsis (18). We postulated that DNA methylation profiling of PBMCs might reveal similar markers in IAP children that could be harnessed to develop endotype signatures. One particularly attractive feature of the ‘epigenetic scars’ concept is the potential to restore functionality using adjuvants that reverse specific nucleic acid base modifications (19, 20). As part of the mission of the National Institutes of Health (NIH)/National Insitute of Allergy & Infectious Diseases (NIAID) Immune Development in Early Life (IDEAL) program, we sought to identify modifiable pathways of immune development with therapeutic potential by investigating the impact of a common adjuvant on endotype-specific molecular signatures. This approach may identify molecules with the potential to redirect the course of immune development in vulnerable children away from endotypes associated with disease and towards those associated with health (21).

In this proof-of-concept study, we retrospectively selected children from a longitudinal child cohort enrolled and followed prospectively in Rochester from 6 to 60 months of age. PBMCs from 14 IAP infants collected in the first year of life were matched to 16 non-respiratory infection allergy/asthma-prone (NIAP) controls using extremes of phenotype design. Sample classifications for vaccine responsiveness determined from antibody responses to primary vaccinations were previously available on this cohort (22). PBMCs collected from these children, were cultured with vehicle control or with monophosphoryl lipid A (MPL) which is a TLR4 agonist. At culture endpoint, cells were harvested for methylome- and genome-wide association analysis using Infinium microarray technology. Using differential analysis, we identified an endotype signature of epigenetic differences in unstimulated cells that was partially modifiable using MPL adjuvant following *in vitro* stimulation

## Results

### Study Participant Characteristics

The characteristics of the participants are presented in Table 1. The IAP group consisted entirely of Caucasian ancestry, whereas the NIAP group comprised 81% Caucasians and 19% other ethnicities. Principal component analysis (PCA) of genotypes confirmed that the study cohort was predominantly of Caucasian ancestry, with three individuals clustering with middle clines reflecting Latino and African American ancestry components (**Figure S1**). The IAP group were enriched with the low vaccine responder (LVR) phenotype (Fishers Exact p-value = 0.01) and had a higher burden of stringently defined otitis media (Fisher’s Exact p-value = 3.3×10^− 5^).

### Epigenome-Wide Association Study of Endotype-Linked Methylation Patterns

**Figure 1:**
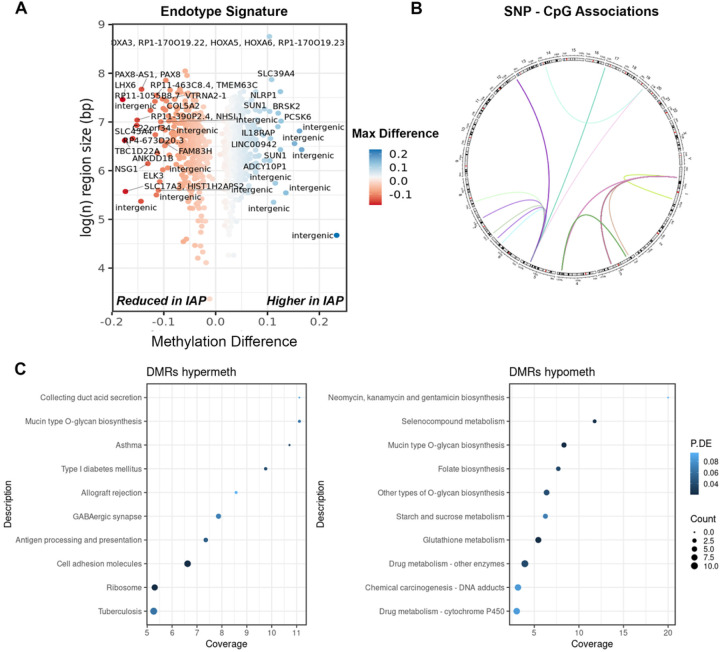
Epigenome-wide association study of IAP and NIAP infants demonstrates differentially methylated regions enriched with immune response genes. (A) Volcano plot of differentially methylated regions significantly associated with IAP status. X-axis represents the average methylation change from NIAP group (delta methylation ratio) and the y-axis shows the log region size in base pairs. Points are coloured according to maximum observed methylation difference in region. (B) Circular visualization of significant SNP-CpG association pairs showing chromosomal location of associated markers. (C) Boxplot of representative methylation quantitative trait loci (mQTLs). Y-axis shows mean methylation ratio with standard deviation stratified by SNP genotype. (D) Dotchart of summary statistics from gene ontology enrichment analysis of the infection prone associated methylation signature highlighting significantly enriched pathways. The y-axis shows gene set nomenclature, point size reflects total count of differentially methylated genes, points are ranked by the percentage of genes in the set covered, and coloured according to false discovery rate adjusted P-value. *Diff = Differentially, DMRs = differentially methylated regions, IAP = Infection allergy/asthma prone, NIAP = non-infection allergy/asthma prone, PBS = phosphate buffered saline, max = maximum, bp = base pairs, P.DE = p-value for over-representation of the GO or KEGG term, Coverage = percentage of genes identified within specified pathway*.

We conducted an epigenome-wide association study (EWAS) comparing unstimulated mononuclear cells from IAP infants vs. NIAP infants. This analysis identified 813 differentially methylated regions (DMRs) encompassing 268 lead CpG associations with genome-wide significance (Fig. 1A). Notably, most regions exhibited higher methylation (hypermethylation) in the IAP group (60.4%, 491 regions).

To assess the role of genetic variation in shaping this endotype-associated methylation signature, methylation quantitative trait loci (mQTL) analysis was performed using individual regression models for SNP-CpG pairs within a +/−500 kb window of endotype-associated CpGs (1,079,685 SNP markers). This analysis revealed 52 unique SNP associations with methylation levels at 5 CpG dinucleotides (FDR < 0.05) (Fig. 1B).

Gene ontology analysis showed enrichment for genes involved in mucin-type O-glycan biosynthesis in both hyper- and hypomethylate DMRs (Fig. 1D). Hypermethylated regions contained genes like *B3GNT3, B3GNT6, GALNT9, ST3GAL1, FUT11*. Hypomethylated regions included *GALNT2, GALNT18, GALNT9*, suggesting potential links to epithelial dysfunction (23, 24). Notably several relevant disease disease pathways including asthma, diabetes, graft rejection, and antigen processing pathways were enriched in hypermethylated regions. The latter involved genes within the major histocompatibility complex (class I member *HLA-F*, class II members *HLA-DPA1* and *HLA-DPB1*and protease subunit *PSMB8*) (25–27).

Additional hypermethylation was observed in genes associated with the inflammasome pathway in IAP children, including *PCSK6* (28), *NLRP1* (29), *FLT4* and *IL18RAP* (30) (31), suggesting deficits in anti-viral defenses. Furthermore, hypermethylation was also observed in immune response genes including *MAP3K6, KSR1* (32), and *PARP9* (33), implicated in the IFN-γ response. *AKT1* involved in the PI3K/AKT/mTOR pathway (34), and *MYD88*, crucial for TLR signalling also exhibited hypermethylation (35). Conversely, hypomethylated DMRs were primarily enriched with genes involved in cellular metabolism, such as glutathione metabolism (*GCLC, GSTA4*, and *GSTM3*) (36), and starch and sucrose metabolism (*HK2* and *PYGB*) (37, 38).

To decipher the cellular specificity this endotype signature, immune cell subset deconvolution of peripheral blood methylation profiles was performed using differentially methylated cell type (DMCT) analysis (39). This analysis did not reveal differentially methylated cell types, suggesting a generalized signature across immune cell subsets (**Figure S2**).

### In vitro MPL stimulation Alters DNA Methylation in Endotype-Associated Genes

To explore how innate immune activation might affect disease-linked methylation patterns, PBMC from the same subjects were stimulated with MPL for 24 hours and analyzed via EWAS. Compared to resting cells, MPL stimulation induced modest methylation changes (average effect size ~ +/− 5%) in 113 regions (FDR < 0.05), encompassing both hypermethylation (60 regions) and hypomethylation (53 regions). Immune cell type distributions remained unchanged after stimulation (**Figure S2**).

**Figure 2:**
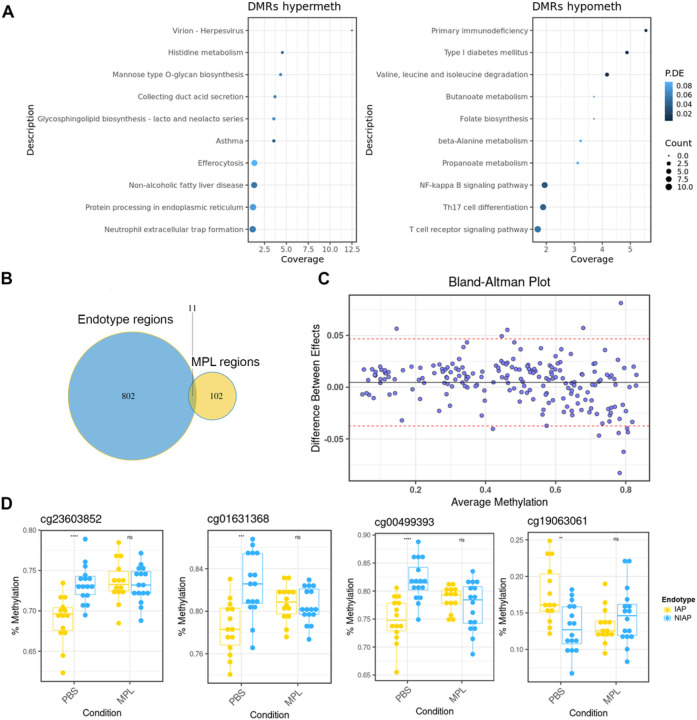
Stimulation with MPL adjuvant modifies a subset of endotype-associated CpGs. (A) Dotchart of summary statistics from gene ontology enrichment analysis for the MPL-associated differentially methylated regions highlighting significantly enriched pathways. The y-axis shows gene set nomenclature, point size reflects total count of differentially methylated genes, points are ranked by the percentage of genes in the set covered, and coloured according to false discovery rate adjusted P-value. (B) Venn diagram of overlapping regions showing counts of unique and overlapping regions. (C) Bland-Altman visualization of methylation difference after MPL stimulation for 183 MPL-sensitive CpGs. Y-values represent the difference in log2 fold-change (NIAP – IAP) between PBS and MPL condition. X-axis shows mean methylation value for each CpG. Upper and lower 95% confidence intervals are shown as red lines. (D) Boxplot of median and quartile range for the four most significantly ranked endotype-associated CpGs modified by MPL treatment. DMRs = differentially methylated regions, MPL = monophosphoryl lipid A, P.DE = p-value for over-representation of the GO or KEGG term, Coverage = percentage of genes identified within specified pathway. Hypermeth = hypermethylated regions. Hypometh = hypomethylated regions.*** = P<0.001, **=P<*0.05 – 0.05, ns= P* > *0.05, two sided t-test*.

Gene ontology analysis revealed enrichment of similar pathways as the endotype signature (eg, asthma, type 1 diabetes) and additional pathways linked to inflammasome signaling (NF-κB signaling, Th17 cell differentiation) suggesting the potential of MPL to target endotype-associated methylation (Fig. 2A). A modest but significant overlap (P = 0.002 hypergeometic test) between endotype- and stimulation-associated regions was detected (Fig. 2B). Interestingly, 183 of 268 significant CpGs differentiating NIAP and IAP groups lost significance after MPL treatment (FDR P ≥ 0.05), indicating specific methylation pattern modulation by MPL.

Bland-Altman visualization (Fig. 2C) confirmed effect size modulation on inter-group methylation variability. Notably, 8 CpGs displayed > 5% modulation of the IAP-NIAP group difference by MPL stimulation (FDR P < 0.05, interaction test, Fig. 2D).

## Discussion

This study introduces a novel approach to identifying children vulnerable to respiratory infections and low vaccine responsiveness through precision epigenetic endotyping. This approach holds potential to revolutionize early detection and guide targeted interventions, ultimately improving immune resilience and preventing chronic disease in this at-risk group (5). Aligning with the NIH/NIAID’s IDEAL consortium mission, our work aims to personalize immunizations and prevent infectious diseases in early life by combining disease- and adjuvant-associated molecular signatures to identify molecules capable of reversing disease pathways. These results pave the way for larger-scale validation studies with the hope of personalized immunotherapies, transforming healthcare for children at high risk of severe respiratory illnesses.

Children within this long-term Rochester cohort experiencing a high burden of recurrent infections in their first five years of life (5) exhibited a distinct molecular signature in mononuclear immune cells collected during infancy. The endotype-signature was characterized by increased methylation in genes governing pro-inflammatory immune responses including inflammasome formation, antigen processing, and glycan biosynthesis. This suggests a potential role in heightened susceptibility to respiratory infections (23, 40). While our analysis indicated minimal influence from genetic risk variants, implying the disease signature reflects more of a developmental programming element, further studies with larger sample sizes are needed to solidify this conclusion.

Notably, this study demonstrates the ability of our methylation profiling approach to identify clinically relevant signatures that corroborate existing clinical and immunological findings in IAP children (2, 4, 7, 8, 10, 41–44). For example, we observed increased methylation at several MHC genes, consistent with the previous reports of reduced surface expression of MHC II proteins in this population (2). Additionally, our findings regarding increased methylation in *MyD88*, a key component of PRR-mediated signalling, align with previous observations of reduced IRF7 mRNA expression, and IFN- α production (2, 9–11). Similarly, increased methylation of inflammasome-related genes (*NLRP1, IL18RAP, PCSK6, FLT4*) in IAP children is consistent with reduced IL-1β mediated T-helper 17 immunity reported in children prone to AOM (9). Future large-scale investigations incorporating functional assays are curcial to validate these observations and explore their potential for personalized interventions in vulnerable children.

While increased methylation at identified genes suggests potential dysregulation in relevant pathways, the functional consequences require further investigation. Hypermethylation often impedes DNA accessibility, potentially reducing gene expression and contributing to the IAP phenotype (45). However, the context-dependent nature of methylation necessitates additional functional follow-up studies. Importantly, it is critical to recognize that the observed changes may not reflect causal drivers but could also arise from gene-environment interactions or disease manifestations.

While our findings reveal minimal overall overlap between endotype- and stimulation-associated methylation patterns, the selective modulation of 8 CpGs by MPL suggests its potential to fine-tune methylation landscapes and potentially influence downstream transcriptional and functional pathways. Further investigations into these specific CpGs are warranted to elucidate the precise mechanisms underlying MPL’s functional impact. The results reported here encourage further development of this approach, leveraging a platform of *in vitro* modelling combined with single-cell epigenetic analysis to relate adjuvant compounds to their target genes. Developing a computational epigenetic signatures database could provide a valuable resource for precision immunisation approaches in vulnerable populations and a starting point for pre-clinical studies. This study establishes the foundation for a precision epigenetic endotyping approach, potentially improving health outcomes in children at high risk for respiratory illnesses.

## Methods

### Sample Selection

Participants in a prospective trial conducted in Rochester NY, USA were children recruited from community-based primary care pediatric practices, as previously described (5). Infection prone status was determined from a data-driven analysis of illness visits over the first 5 years of life, described in (6). For the current study, a subset of 30 participants was selected, 14 of which were IAP cases and 16 NIAP controls. Participant selection was intentionally guided to emphasize the extremes of clinical phenotypes, that is, respiratory infection allergy/asthma-prone children (IAP) enriched for low vaccine responder (LVR) phenotype vs. non-respiratory infection allergy/asthma prone (NIAP) (Table 1) (22). The study ethics and protocol were approved by The Rochester Regional Health Human Subjects Review Board under the approval number CIC 1141-B-09-1 (46).

#### PBMC stimulation in vitro

Cryopreserved PBMCs were thawed by dropwise addition of cold RPMI 1640 medium (Gibco) supplemented with penicillin/streptomycin (Gibco), 2 mM l-glutamine (Gibco), and 10% bovine serum (Hyclone). Stimuli included synthetic Monophosphoryl Hexa-acyl Lipid A, 3-Deacyl (3D(6A)PHAD, Avanti Polar Lipids), abbreviated to MPL. MPL was used at a concentration of 1 μg/mL. MPL was prepared at 10X the desired concentration and 20 μL was added to flat-bottom sterile, pyrogen-free 96-well culture dish wells (Corning). PBMCs were washed with RPMI, counted, and viable cells determined by Trypan Blue staining (Gibco) were resuspended in RPMI supplemented with penicillin/streptomycin, 2 mM l-glutamine, and 10% autologous plasma at a concentration of 2.78 million cells/mL. 180 μL of cell suspension (500.000 PBMCs) were plated on top of stimuli. Plates were incubated for 24 hours in a humidified incubator with 5% CO2. After 24 hours, culture supernatants were stored at − 80 C and cells were centrifuged for 3 minutes at 500 rpm. Cell pellets were resuspended in 500 μL RNAlater (Sigma-Aldrich) for downstream DNA methylation profiling and frozen at −80 degrees.

### DNA methylation Profiling

PBMC pellets were thawed and washed in phosphate buffered saline solution (PBS) prior to genomic DNA extraction using the Chemagic DNA 400 kit H96 (cat # CMG-1491). DNA samples were quantified using the Qant-iT HS kit (cat# Q33120) and randomized by endotype label prior to being sent to the Australian Genome Research Facility in Melbourne, Western Australia, for bisulfite conversion and genotyping using Illumina Infinium MethylationEPIC Beadchip v1 arrays. Bisulfite-converted genomic DNA was analyzed using Illumina’s Infinium Human Methylation EPIC BeadChips, which enable methylation measures at over 850,000 CpG sites. Raw.iDAT files were pre-processed using the Min (47) package from the Bioconductor project (http://www.bioconductor.org) in the R statistical environment (http://cran.r-project.org/version 4.2.2). Sample quality was assessed using control probes on the array, and no samples were removed. Array normalization employed the stratified quantile method to correct for type 1 and type 2 probe biases. Probes exhibiting a P-detection call rate of > 0.01 in one or more samples were removed (44,881 probes) prior to analysis. Probes containing SNPs at the single base extension site or at the CpG assay site were removed, as were probes measuring non-CpG loci (30,015 probes). Probes reported to have off-target effects by McCartney et al. (48) were also removed (39,489 probes). After filtering, the final dataset consisted of 120 samples and 751,474 probes. Methylation ratios were derived as β values ((methylated alleles)/((unmethylated + methylated) × 100)) with log 2 transformation to M values for statistical analysis.

### Genotyping and imputation

Aliquots of genomic DNA samples were genotyped by the Australian Genome Research Facility using the Illumina Global Screening Array v3 with a multi-disease drop-in. Genotype calling was performed using the *gencall* algorithm in GenomeStudio (Illumina). Quality control was performed using the plinkQC package (v 0.3.4) (49) to remove samples with > 5% missing data, with high relatedness (PI_HAT > 0.2) in identity-by-descent analysis for all pairs of samples, or with mismatched ancestry estimates based on principal component analysis of merged data with the 1000 genomes phase 3 data set. We also excluded SNPs characterized by > 5% missing values, a Hardy-Weinberg equilibrium p-value < 0.001, and a minor allele frequency of < 5%. Quality controlled data were then imputed with the Haplotype Reference Consortium hg19 r1.1 reference panels using Beagle 5.4 on the Michigan imputation server. Imputed genotypes were filtered to remove SNPs with a minor allele frequency of < 5% and Hardy-Weinberg equilibrium p-value < 0.001, with an r2 value > 0.3.

### Statistical analysis

Linear regression modelling employed the R statistical environment using the limma package (50) to test the association between DNA methylation, IAP status, and stimulation conditions. A factorial model with main effects on disease status and stimulation, including covariate adjustment for cell-type proportions, the 1st five principal components of the methylation data set, 1st two principal components of the SNP data set, sex, and age, was fitted to the methylation ratios (M-values). We used the corfit function to adjust the variance estimates for the repeated-measures samples. To determine endotype-specific signatures, we compared unstimulated samples for the main effect of disease status adjusted for covariates. Summary statistics from the model fit were used to identify differentially methylated regions (DMR) using the R package DMRcate (51). DMRs were defined using a lambda of 1000, min. cpgs of 4, and adjusted p-cutoff of 0.05. To derive stimulus-specific signatures, we compared each stimulation with the unstimulated control samples, adjusting for disease status and covariates. Enrichment analysis of all differentially methylated regions was conducted using the missMethyl R package (52). Cell-type proportions were estimated using the EpiDish (Epigenetics Dissection of Intra-Sample Heterogeneity) package in R (39). The cis-meQTL test for association of nearby SNPs with DNA methylation measurements was carried out using the MatrixEQTL R package (v2.3) (53), with covariate adjustment for sex, and the five principal components of methylation array control probes and genotyping array ancestry estimates.

#### Data management and deposition.

Data management for this project utilized a centralized cloud computing based management approach as previously described (54, 55). Using quality control (QC) procedures for clinical, immunologic and epigenetic data, we implemented rigorous checks to ensure the reliability and accuracy of the datasets. Quality assurance (QA) involved systematic processes to validate the integrity and consistency of the generated data. This included verifying data completeness across datasets, conducting checks for accuracy and ensuring adherence to predefined standards. We also addressed potential issues, such as missing values, outliers and discrepancies.

## Figures and Tables

**Table 1 –:** Demographics of study cohort

Variable	Category	IAP	NIAP	Overall
Vaccine responsiveness				
	High vaccine response	(1) 7%	(5) 31%	(6) 20%
	Normal vaccine response	(6) 43%	(10) 62%	(16) 53%
	Low vaccine response	(3) 21%	(1) 6%	(4) 13%
	Very low vaccine response	(4) 29%	(0) 0%	(4) 13%
Otitis prone status				
	Stringently defined otitis prone	(10) 71%	(0) 0%	(10) 33%
	Non otitis prone	(4) 29%	(16) 100%	(20) 67%
Atopy				
	Eczema	(4) 29%	(3) 18%	(7) 23%
Sex				
	Female	(3) 21%	(6) 38%	(9) 30%
	Male	(11) 79%	(10) 62%	(21) 70%
Age (months)	Mean	9.9 ± 2	10.76 ± 1.59	10.36 ± 1.81
Ancestry				
	African American	(0) 0%	(1) 6%	(1) 3%
	Caucasian	(14) 100%	(13) 81%	(27) 90%
	Other	(0) 0%	(2) 12%	(2) 7%
Daycare				
	Yes	(11) 79%	(8) 50%	(19) 63%
	No	(3) 21%	(8) 50%	(11) 37%
Breastfeeding				
	Breastfed	(4) 29%	(3) 19%	(7) 23%
	Formula fed	(8) 57%	(10) 62%	(18) 60%
	Both	(2) 14%	(3) 19%	(5) 17%
Smokers in home				
	Yes	(0) 0%	(1) 6%	(1) 3%
	No	(14) 100%	(15) 94%	(29) 97%
Siblings				
	Yes	(7) 50%	(11) 69%	(18) 60%
	No	(7) 50%	(5) 31%	(12) 40%

## Data Availability

Following the de-identification and QC/QA processes, the data were securely deposited into ImmPort under accession number SDY2506. All analysis codes have been deposited at Bitbucket https://bitbucket.org/pvp-dmac/ideal_pilot/src/main/ and are publicly available as of the date of publication. Any additional information required to reanalyze the data reported in this paper is available from the lead contact upon request.
